# Suppression of SAMSN1 contributes to neuroprotection in neonatal rats suffering from hypoxic–ischemic encephalopathy injury

**DOI:** 10.1002/ibra.12078

**Published:** 2022-11-12

**Authors:** Yi‐Bo Wang, Zong‐Jin Gan, Jun‐Yan Zhang, Somjit Wanchana, Xi‐Liang Guo

**Affiliations:** ^1^ School of Basic Medical Sciences Jinzhou Medical University Jinzhou Liaoning Province China; ^2^ Class of 2019, Department of Anesthesiology Southwest Medical University Luzhou Sichuan Province China; ^3^ Boromarajonani College of Nursing Ratchaburi Thailand; ^4^ Department of Human Anatomy, School of Basic Medical Science Jinzhou Medical University Jinzhou China; ^5^ Liaoning Key Laboratory of Diabetic Cognitive and Perceptive Dysfunction Jinzhou Medical University Jinzhou China

**Keywords:** SAM domain, SH3 domain and nuclear localization signal 1, cell viability, neonatal hypoxic–ischemic encephalopathy, neurological injury, neuroprotective effect

## Abstract

This article aims to detect the effect of SAM domain, SH3 domain, and nuclear localization signal 1 (SAMSN1) in neonatal rats with neurological dysfunction induced by hypoxia and ischemia (HI). The HI model was created using 7‐day postnatal rats. Zea‐longa score was utilized to validate the neurological injury after HI. Then, the differentially expressed genes (DEGs) were detected by gene sequencing and bioinformatics analysis methods. The oxygen and glucose deprivation (OGD) models were established in the SY5Y cells and fetal human cortical neurons. In addition, SAMSN1‐small interfering RNA, methyl thiazolyl tetrazolium assay, and cell growth curve were employed to evaluate the cell viability variation. Obviously, Zea‐longa scores increased in rats with HI insult. Subsequently, SAMSN1 was screened out, and it was found that SAMSN1 was strikingly upregulated in SY5Y cells and fetal neurons post‐OGD. Interestingly, we found that SAMSN1 silencing could markedly enhance cell viability and cell growth after OGD. These data suggested that downregulation of SAMSN1 may exert a neuroprotective effect on damaged neurons after HI by improving cell viability and cell survival, which provides a potential theoretical basis for clinical trials in the future to treat neonatal hypoxic–ischemic encephalopathy.

## Graphical abstract

The purpose of this study was to investigate the effects of the SAM domain, SH3 domain, and nuclear localization signal 1 (SAMSN1) in neonatal rats with neurological dysfunction induced by hypoxia and ischemia (HI). Differentially expressed genes in the HI model established by Sprague–Dawley rats were detected by gene sequencing and bioinformatics analysis, and HIE‐related SAMSN1 was screened out. SY5Y cells and fetal human cortical neurons were used to establish a cell model of oxygen and glucose deprivation‐induced injury, and the cell viability variation is evaluated by SAMSN1‐small interfering RNA, methyl thiazolyl tetrazolium assay, and cell growth curve. The results suggest that downregulation of SAMSN1 may play a neuroprotective role in HI‐impaired neurons by increasing cell survival rate and cell survival rate.

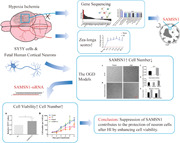

## 1 INTRODUCTION

Neonatal hypoxic–ischemic encephalopathy (NHIE) may lead to death or severe neurologic deficits in neonates. NHIE is one of the main causes of neonatal death because it has a great negative impact on the brain development of newborns, and has the highest mortality and morbidity among infants worldwide. The incidence of HIE in the United States is 2–3 in 1000 fetuses, and 20%–25% of term newborns die in the neonatal period.^1^ Moreover, approximately 25% of survivors suffer from motor, sensory, and cognitive impairment.^2^ Studies have proved that HIE is caused by persistent cerebral hypoxic–ischemic (HI) diseases, which can directly lead to irreversible damage such as neuron necrosis and persistent inflammation.^3^ So far, there have been many methods to treat HIE, such as fluids therapy, antiepileptic drugs, stem cell therapy, and hypothermia therapy,^1^ and mild hypothermia therapy is regarded as a beneficial treatment for children with HIE in terms of reducing brain damage and enhancing neurological function.^4^ Though hypothermia prognosis results seem to be impactful in mild to moderate injuries.^5^ In contrast, it remains to be confirmed whether therapeutic hypothermia can improve the prognosis of children with severe HIE, and practical clinical treatments or preventive measures remain unavailable. Thus, the potential mechanisms and effective treatment of NHIE urgently need to be further investigated.

Although HIE has complex pathophysiological characteristics, the various steps leading to cell damage provide many opportunities for therapeutic intervention. SAM domain, SH3 domain, and nuclear localization signal 1 (SAMSN1), also known as HACS1, NASH1, or SLY2, is a member of a new family of putative adaptor proteins and scaffold proteins containing SH3 and sterile alpha motif (SAM) domains,^6^ which are primarily expressed in hematopoietic cells mediates B‐cell differentiation and activation and located in a region on human chromosome 21 (21q11‐2). Evidence suggests that SAMSN1 is frequently subjected to translocation events in hematopoietic malignancies and is related to the frequent deletions of heterozygotes in lung cancer cells.^7^ In addition, SAMSN1 was found to be closely related to glioblastoma multiforme, and upregulated SAMSN1 was a dangerous factor for patients with glioblastoma multiforme.^8^ In a word, previous studies mainly concentrated on the roles of SAMSN1 in varieties of cancers such as gastric cancer,^7^ multiple myelomas (MM),^9^ colorectal cancer,^10^ hepatocellular carcinomas,^11^ and angioimmunoblastic T‐cell lymphoma,^12^ which indicated that SAMSN1 is closely associated with cancer and immune system disease. Nevertheless, to date, little research investigated the effect of SAMSN1 on HI‐induced neurological defects, and needs further exploration.

This study validated neurological damage in neonatal rats after HI using the Zea‐longa scores test. Then, SAMSN1 was screened out, and its function in developing NHIE was analyzed by bioinformatics analysis. Additionally, the effects of SAMSN1 on SY5Y cells and fetal human cortical neurons were further investigated with SAMSN1‐small interfering RNA (siRNA), 3‐(4,5‐dimethylthiazol‐2‐yl)‐2,5‐diphenyltetrazolium bromide (MTT) assay, and cell growth curve following oxygen and glucose deprivation (OGD). For the first time, the present study reported the close connection between SAMSN1 expression and HIE and provided a foundation for the molecular pathogenesis of NHIE in clinical trials.

## 2 MATERIALS AND METHODS

### 2.1 Animals and grouping

A total of 20 SD rats (7 days old, weighing 12–15 g) were purchased from the Animal Center of Kunming Medical University and fed following the guideline of the China Laboratory Animal Protection and Ethics Committee. The study was approved by the Ethics Committee of Kunming Medical University and the Animal Care & Welfare Committee of Kunming Medical University (no. KMMU2020001) and followed the Guide for the Care and Use of Laboratory Animals published by the US National Institutes of Health. The rats were kept in 45%–50% humidity, 12‐h light/dark alternating mode, the room temperature was maintained at 21–25°C, and they ate and drank freely. Neonatal rats were randomly divided into the sham and HI groups (*n* = 10/group).

### 2.2 Establishment of HI rat models

Our HI rat models were established based on the classic Rice‐Vannucci HI models.^13^, ^14^ The 7‐day‐old rats were anesthetized with 3% isoflurane (RWD) and fixed on the operating table. Then, the right common carotid artery was separated and exposed, followed by permanent burning of the right common carotid artery using a monopolar microsurgery electrocoagulator (Spring Medical Beauty Equipment Co.). After the rats regained consciousness, they were removed into a chamber filled with oxygen (8%) and nitrogen (92%) at 37°C for 2 h to induce the hypoxia. The right common carotid artery in the sham group was just exposed without separation.

### 2.3 Zea‐longa scores

The blinded Zea‐longa scale score was applied before and after surgery to evaluate the neurological functions.^15^ All neurological tests were assessed by researchers not involved in this study.

### 2.4 Sample collection and gene sequencing

At 24 h after HI modeling, the neonatal rats in the sham group (*n* = 10) and the HI group (*n* = 10) were anesthetized with 3% isoflurane (RWD). The heart was perfused with normal saline until the liver turned white. An incision was made in the skin and skull at the top of the brain, the brain was exposed, and the brain was removed and placed in 0.1 M precooled phosphate‐buffered saline (PBS). After the pia mater and blood vessels were entirely removed, the right cerebral cortex tissues were carefully removed. Total RNA was extracted from the cortical tissue surrounding the cerebral infarction site. The cerebral tissues were collected according to the manual and the gene sequencing was carried out by Biomarker Technologies (Beijing, China).

### 2.5 Bioinformatics analysis

The differentially expressed genes (DEGs) were analyzed using gene ontology (GO) and Kyoto encyclopedia of genes and genomes (KEGG) analysis, and STRING online tools (https://string-db.org/cgi/input.pl). GO analysis was employed to annotate biological processes, molecular function, and cellular components.

KEGG systematic analysis was used for routine analysis of gene functions and related higher‐order genomic functional information of SAMSN1. Here, GO and KEGG analyses were applied to decipher the primary molecular functions and signaling pathways of DEGs. Besides, the SAMSN1‐associated protein network was constructed by String.

### 2.6 Culture of SY5Y cells

SY5Y cell lines (GeneCopoeia Company) were cultivated in flasks with a 1:1 mixture of Dulbecco's modified Eagle's medium (DMEM) (1.5 g/L sodium bicarbonate, 2 mM l‐glutamine, 1 mM sodium pyruvate, and 0.1 mM nonessential amino acids), Ham's F12 medium, and 10% supplemental fetal bovine serum (FBS) at 37°C with 5% CO_2_. Cells were seeded (0.5 × 10^4^ cells/cm^2^), and then cultured at 37°C with 5% CO_2_. SY5Y cells were passaged after 2 days in culture and then used for the establishment of the OGD model.

### 2.7 Culture of fetal human cortical neurons

The cortical brain tissues were harvested from a naturally aborted fetus (29 days old) provided by the First Affiliated Hospital of Kunming Medical University, which was approved by the Ethics Committee of Kunming Medical University on September 30, 2015 (approval number: 2015‐9). After being cut into 1 mm^3^ tissue blocks in the pre‐cold DMEM, the cells from cortical tissues were isolated by using 0.25% trypsin at 37°C for 30 min. Following elution with 10% FBS (Hyclone), the obtained suspension was centrifuged (1000 rpm) for 10 min. The pellets at the bottom were subsequently resuspended in the complete culture medium (Hyclone) containing DMEM/high glucose (Hyclone), 10% FBS, and 1% penicillin–streptomycin solution (Hyclone). Viable neurons were counted by Trypan blue exclusion and then seeded into 96‐well plates (Corning) with substrate pretreatment at 5 × 10^5^ cells/ml, followed by incubation at 37°C with 5% CO_2_. The medium was replaced with a neurobasal medium with 2% B27 (Invitrogen) after 4 h and was changed the next day. Half of the medium was changed every 3 days.

### 2.8 Establishment of OGD model

SY5Y and fetal human cortical neurons were washed with PBS and then placed into the glucose‐free medium (Gibco) at 37°C. Afterward, the cells were placed in a chamber filled with 95% N_2_ and 5% CO_2_ at 37°C for 2 h. Subsequently, the cells were re‐placed into a normal medium containing 95% air and 5% CO_2_ for reoxygenation for 24 h.^13^

### 2.9 Real‐time quantitative polymerase chain reaction (RT‐qPCR)

Total RNA was extracted from cells using Trizol reagent (Takara Bio Inc.) according to the manufacturer's protocol strictly and then reversely transcribed to complementary DNA (cDNA) with the Revert AidTM First Strand cDNA Synthesis Kit (Thermo Fisher). Primer sequences were designed by Primer 5.0 software for RT‐qPCR as follows: SAMSN1: forward, 5′‐CACTGCTGTCCGTTCTCC‐3′; reverse, 5′‐GCTCTTCCTTCTTACCCTCA‐3′; GAPDH: forward, 5′‐TGACTTCAACAGCGACACCCA‐3′; reverse, 5′‐CACCCTGTTGCTGTAGCCAAA‐3′. Next, the reaction was completed in a DNA thermal cycler (ABI 7300; Thermo Fisher), the process was as follows: one cycle of 95°C for 2 min; 40 cycles of 95°C for 15 s, annealing for the 20 s, and 60°C for 40 s. Data were counted through the normalization of GAPDH values through the $2^{-\Delta \Delta C_{t}}$ method.

### 2.10 Screening effective interference fragments of siRNAs

Gene database of SAMSN1 from the National Center for Biotechnology Information was firstly obtained, and then three siRNAs inhibiting SAMSN1 expression and one noninhibitory and Cy3‐labeled siRNA as negative control (NC) were designed and acquired from Ribobio Company. Briefly, when the fusion rate of SY5Y cells was approximately 40%, a fresh medium containing siRNA fragments or reagents was added. According to the different treatments, SY5Y cells were randomly divided into different groups, including the normal, NC, Reagent, SAMSN1‐F1, SAMSN1‐F2, and SAMSN1‐F3 groups. After 48 h, RT‐qPCR was performed to identify the interference efficiency, and the siRNA fragment with the highest interference efficiency was selected for subsequent experiments.

### 2.11 Target interfering RNA transfection

After culturing SY5Y cells for 5 days, the culture medium was removed in half, and a mixture solution (60 μl 1× buffer, 5 μl siRNA, and 5 μl siRNA reagent) was added to each well of 6‐well plates. The cells were incubated for 15–30 min at normal temperature, adding 1 ml medium per well after 24 h. Images were collected via Leica AF6000 cell station (Leica) after 48 h, and cell numbers were calculated by choosing five randomly selected fields (200×). And the jamming effects were assessed by RT‐qPCR after 4 days of transfection.

### 2.12 MTT assay

Cells were seeded into 96‐well plates and cultured in an incubator. When the cell monolayer was covered with the bottom of the well, 20 μl of MTT solution was added to each well of the 96‐well plate, and the culture medium with MTT was incubated at 37°C for 4 h, and then the medium with MTT was aspirated. Each well was added with 100 μl dimethyl sulfoxide (Biosharp) solution, and then placed the 96‐well plate on an oscillator for 10 min. Finally, cell viability was calculated by and measured the absorbance value at 570 nm using a microplate reader.

### 2.13 Cell growth curves

Cells were seeded in 96‐well plates, images of nine fields were scanned for each time point, and then cells in these images were counted. Each experimental group had three multiple wells, and the cell count values of each group were averaged. With the cell number as the ordinate and time point as the abscissa, the cell growth curve of each group for 5 consecutive days could be drawn. The differences in cell growth curves among the experimental groups could directly represent the differences in cell proliferation and apoptosis.

### 2.14 Statistical analysis

Data were statistically analyzed utilizing SPSS 21.0 software (SPSS) and displayed as the mean ± SD. Independent‐sample *t*‐test was used for comparison between the two groups, one‐way analysis of variance was used for comparison between the three groups or more, least significant difference was used for comparing the difference between the two groups if the variance was homogeneous, Dunnett T3 was used for pairwise comparison if the variance was not homogeneous. *p* < 0.05 was regarded as statistically significant.

## 3 RESULTS

### 3.1 HI‐induced neurological injury in neonatal rats correlated with SAMSN1

**Figure 1:**
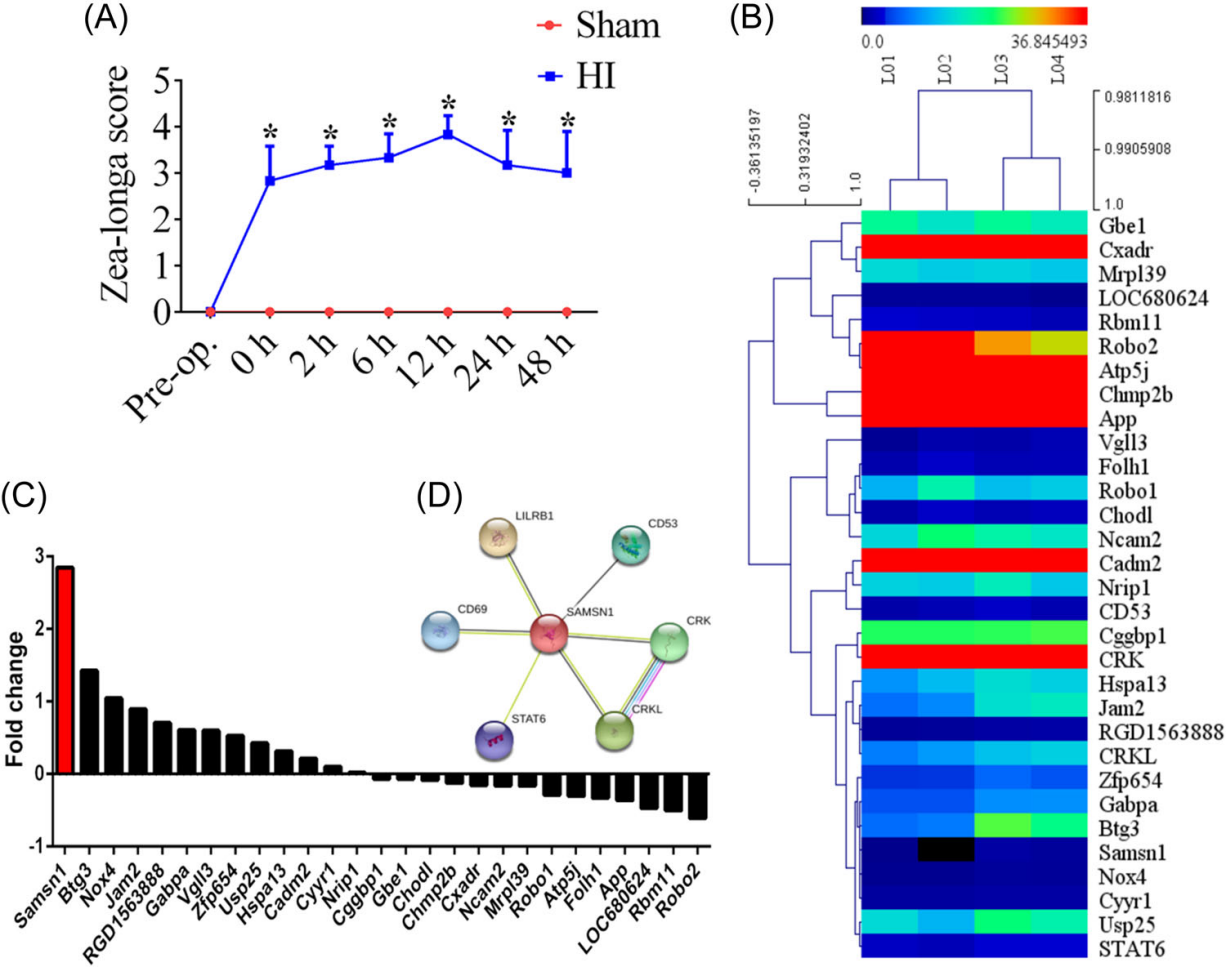
Expression of SAMSN1 in neonatal rats post‐HI. (A) Zea‐longa score of rats from HI and sham groups at 0, 2, 6, 12, 24, and 48 h post‐HI. (B) Heat map and hierarchical clustering analysis of 31 genes differentially expressed in rat cortex between the HI (L03, L04) and sham (L01, L02) group. A color tag represents gene expression level; the red spectrum denotes upregulated genes and green for downregulated ones. (C) Quantitative histograms of the fold change of DEGs. (D) Gene network map between SAMSN1 and related genes. h, hours; HI, hypoxia and ischemia. Data are presented as the means ± SD. *p < 0.05. [Color figure can be viewed at wileyonlinelibrary.com]

The HI brain injury models were established, and the Zea‐longa scores were used to detect nerve damage after HI brain injury in neonatal rats. The results demonstrated that HI brain injury has caused neurological dysfunction. Before the operation, there was no significant difference in Zea‐longa neurological deficit scores between the sham group and the HI group (*p* > 0.05). However, the scores were significantly elevated after HI compared with the sham group, especially at 12 h. A significant decrease in the score was also observed 24–48 h after HI (Figure 1A, *p* < 0.05). To assess the underlying role of HI‐induced injury, gene sequencing was performed to detect the DEGs in HI neonatal rats (Figure 1B). Thirty‐one genes were filtered out, including 17 upregulated genes and 14 downregulated genes. Additionally, we found that SAMSN1 was obviously upregulated in comparison to other genes in the HI group (Figure 1C). Furthermore, the STRING result showed the mutual relationship among DEGs, and SAMSN1 was closely correlated with LILRB1, CD69, CD53, STAT6, CRKL, and CRK (Figure 1D).

### 3.2 GO and KEGG functional enrichment analysis for the DEGs

**Figure 2:**
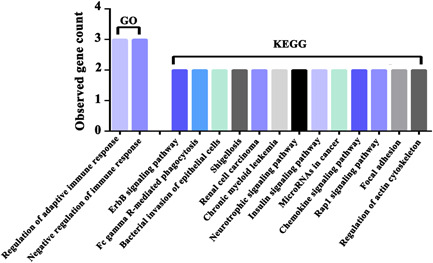
Bioinformatics analysis of SAMSN1. The GO and KEGG pathway analysis of DEGs using the DAVID website. GO, gene ontology; KEGG, Kyoto encyclopedia of genes and genomes pathway analysis. [Color figure can be viewed at wileyonlinelibrary.com]

GO analysis was used for revealing the specific biological function of DEGs, and the result showed that they were mainly involved in the regulation of adaptive immune response and negative regulation of immune response (Figure 2). In KEGG pathway analysis, the main pathways significantly affected were closely related to immune response and cancer, including the ErbB signaling pathway, Fc gamma R‐mediated phagocytosis, bacterial invasion of epithelial cells, shigellosis, renal cell carcinoma, chronic myeloid leukemia, neurotrophic signaling pathway, insulin signaling pathway, microRNAs in cancer, chemokine signaling pathway, Rap1 signaling pathway, focal adhesion, and regulation of actin cytoskeleton (Figure 2).

### 3.3 Expression of SAMSN1 was increased after OGD

**Figure 3:**
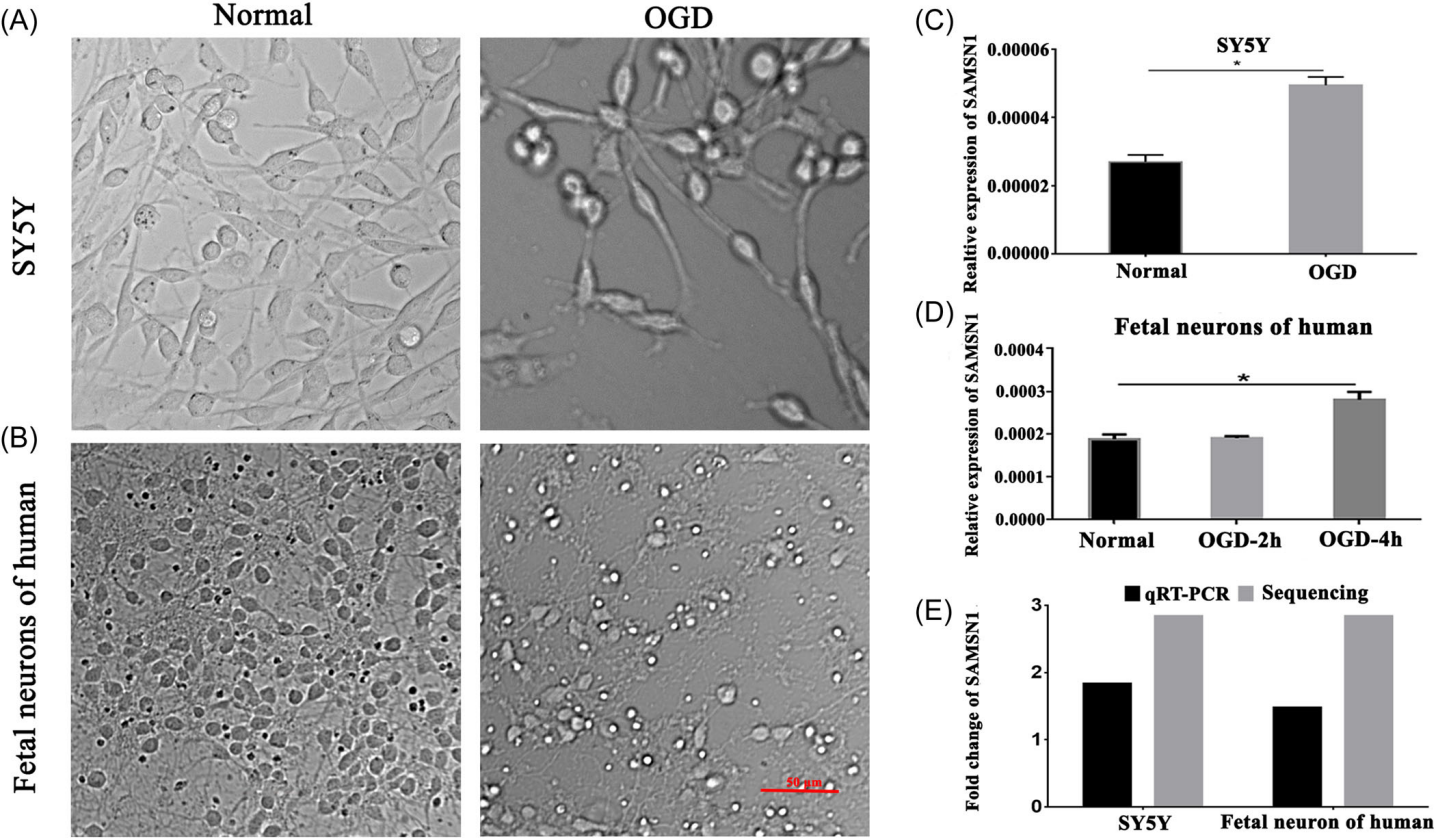
Expression of SAMSN1 post‐OGD. (A, B) Morphology of SY5Y cells and cortical fetal human cortical neurons after OGD under a light microscope, respectively. Scale bar = 50 μm. (C, D) Relative expression changes of SAMSN1 in SY5Y cells and cortical fetal human cortical neurons post‐OGD. *n* = 3/group in cortical fetal human cortical neurons. (E) Fold change of SAMSN1 in SY5Y cells and cortical fetal human cortical neurons. OGD, oxygen‐glucose deprivation. *p < 0.05. [Color figure can be viewed at wileyonlinelibrary.com]

Morphology of SY5Y cells and cortical fetal human cortical neurons s was observed in the normal group and found cone‐shape or multipolar with regular contours. After the OGD insult, the number of cells and neurons showed a notable reduction, and the morphology became slender, round, and degenerated cell debris (Figure 3A,B). Furthermore, the RT‐qPCR was applied to validate the expression of SAMSN1. As a result, SAMSN1 in SY5Y cells after OGD was remarkably increased compared with the normal group (Figure 3C, *p* < 0.05). Moreover, compared with the normal group, the SAMSN1 level in cortical fetal human cortical neurons was significantly enhanced in the OGD‐4 h group (Figure 3D, *p* < 0.05). The fold change of SAMSN1 in cells and neurons showed the same trends with the RT‐qPCR and sequencing results (Figure 3E).

### 3.4 Successful screening of effective interference fragment

**Figure 4:**
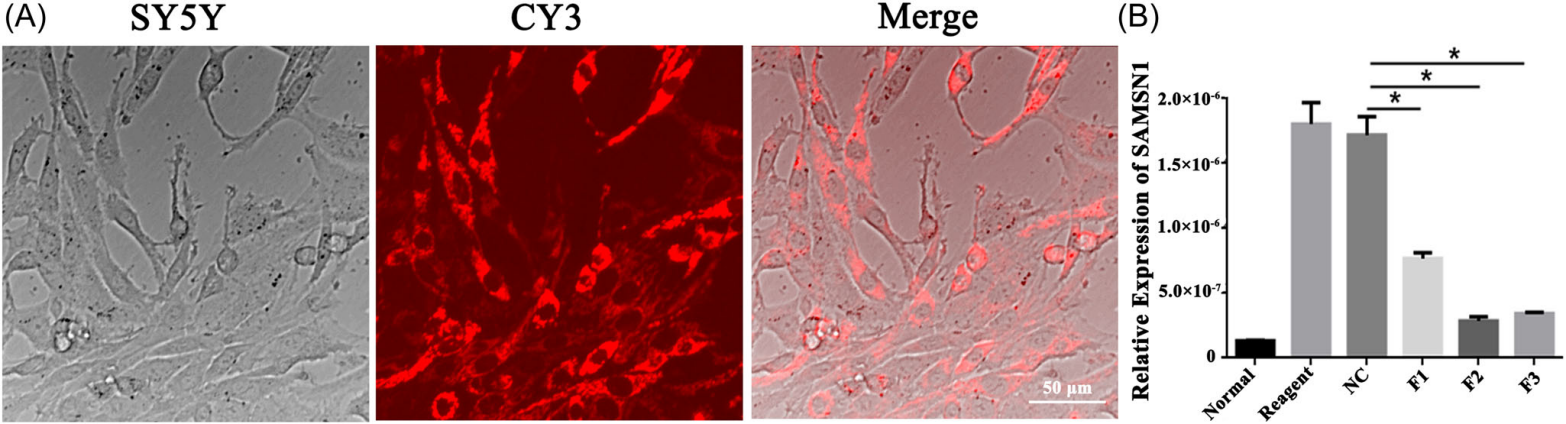
Transfection of SAMSN1‐siRNA into SY5Y cells. (A) SY5Y cells were transfected by CY3 (red) labeled SAMSN1‐siRNA and then collected by a fluorescent microscope. (B) Relative expression changes of SAMSN1 after transfection in normal, reagent, NC, F1, F2, and F3 groups. F1, fragment 1; F2, fragment 2; F3, fragment 3; NC, negative control; SAMSN1‐siRNA, small interfering SAMSN. Scale bar = 50 μm, *p < 0.05. [Color figure can be viewed at wileyonlinelibrary.com]

We further evaluated the role of the F1–F3 interference sequences of SAMSN1 in vitro. The differentially relative expression among groups of normal, reagent, NC, F1, F2, and F3 reflected the extent to which the expression of SAMSN1 was downregulated. Meanwhile, the Cy3 labeled siRNAs were transfected into SY5Y cells and displayed red fluorescence expression in cells, manifesting the SAMSN1‐siRNA efficiently transfected into the SY5Y cells (Figure 4A). In addition, RT‐qPCR results further showed that three siRNAs could downregulate the level of SAMSN1 in contrast to the NC group, and the F2 interference fragment was more effective than the other two (Figure 4B, *p* < 0.05).

### 3.5 Cell viability was improved after SAMSN1 interference

**Figure 5:**
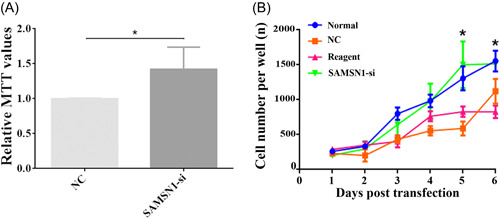
Cell viability and cell number in neurons after transfection. (A) Line chart of relative MTT values in the NC and SAMSN1‐siRNA groups. (B) Cell growth curve exhibited the cell number variation in normal, NC, reagent, and SAMSN1‐siRNA groups. MTT, methyl thiazolyl tetrazolium assay; NC, negative control; SAMSN1‐siRNA, small interfering SAMSNI. *p < 0.05. [Color figure can be viewed at wileyonlinelibrary.com]

MTT assay was used to detect cell viability after SAMSN1‐siRNA transfection. It was found that cell viability was obviously increased after the treatment of SAMSN1‐siRNA compared with the normal group (Figure 5A, *p* < 0.05). Besides, the cell growth curve displayed that cell numbers exhibited an uprising trend 1–6 days after SAMSN1‐siRNA transfection, significantly increasing at 5 and 6 days in comparison to the Reagent group (Figure 5B, *p* < 0.05).

## 4 DISCUSSION

In the current study, the model of HI was successfully established and found that SAMSN1 was obviously increased post‐OGD. In addition, after interfering with SAMSN1, the vitality and number of cells increased significantly. These findings indicated that inhibition of SAMSN1 may further promote the growth of neurons, reduce HI‐induced brain injury, and proved to be a new therapeutic target for NHIE.

### 4.1 SAMSN1 exerts a crucial role in brain injury

The Zea‐longa neurological deficit score was increased in neonatal rats with HI injury. Additionally, in vitro experiments found that the number of neurons was observably decreased in SY5Y and cortical fetal human cortical neurons with OGD, which is in essential agreement with the previous study reporting HI could induce related neuronal injury.^16^ Accumulating evidence shows that SAMSN1 is differentially expressed in many tumors and is related to tumor progression and prognosis.^8^, ^9^ Meanwhile, the deletion of SAMSN1 significantly affected multiple cell types contributing to MM development.^14^ In addition, recent research demonstrated that SAMSN1 is a biomarker crosstalk gene between periodontitis and venous thromboembolism.^17^ Another study has found that platelet gene expression profiling can detect the early signal of impending acute myocardial infarction (AMI) before the occurrence of infarction, and SAMSN1 is one of the DEGs in platelet gene expression profiling^18^; meanwhile, SAMSN1 is reported as a gene associated with coronary atherosclerosis and B‐cell activation.^19^ In the nervous system, SAMSN1 was related to cerebral malaria by using genomic analysis of host gene responses to cerebral *Plasmodium falciparum* malaria.^20^ Moreover, in Alzheimer's disease, using GWAS, SAMSN1 was found to be below the genomewide significance threshold, and it was significantly upregulated when exposed to amyloid β‐protein.^21^ Meanwhile, we found that SAMSN1 was significantly increased post‐HI injury, as well as increased in SY5Y cells and cortical fetal human cortical neurons subjected to OGD, while silencing SAMSN1 could improve cell viability and promote cell growth. The above studies indicate that SAMSAN1 exerts a vital role in brain injury diseases and may be a potential and key target for future brain injury research.

### 4.2 Prediction of target genes associated with SAMSN1 in NHIE

To further explore the regulatory mechanism of SAMSN1 in HIE, LILRB1, CD53, CRK, CRKL, STAT6, and CD69, which were found to be correlated with SAMSN1 according to STRING. LILRB1, a member of the leukocyte immunoglobulin‐like receptor (LILRB) family, is thought to control inflammatory responses and cytotoxicity to help focus the immune response and limit autoreactivity. It has been reported that inhibitory LILRBs play an essential role in innate immunity.^22^ Besides, LILRB1 is related to the pathologic progression of varieties of cancers like adenocarcinoma,^23^ hepatocellular carcinomas,^24^, ^25^ pancreatic cancer,^26^ and so on. CRK, located on chromosome 17P13.3, encodes a signaling protein involved in cell proliferation, differentiation, migration, and axon growth. When microdeletions or microduplications occur, CRK influences neuronal migration and contributes to the development of neurodevelopmental genetic diseases.^27^ CRKL, a substrate of the BCR‐ABL tyrosine kinase involves in fibroblast transformation by BCR‐ABL and may be oncogenic. A previous study suggested that miR‐429 may act as an antimetastatic microRNA to adjust HCC metastasis by directly targeting CRKL through modulating Raf/MEK/ERK‐EMT pathway.^28^ Furthermore, CD53,^29^ CD63,^30^ and STAT6^31^ take part in the regulation of cell development, growth, and activation. GO and KEGG functional enrichment analysis indicated that regulation of adaptive immune response and negative regulation of immune response is involved in the formation of HIE. The activation of innate immunity and the release of inflammatory mediators are important factors in reperfusion injury involving microglia.^32^ The neurotrophic signaling pathway is involved in the protection of δ‐opioid receptors and histone acetylation against HI.^33^, ^34^ The insulin signaling pathway is essential for growth hormone and insulin‐like growth factor‐1 to exert protective effects on HIE.^35^ However, the relative regulatory mechanism of these target genes and pathways needs to be further investigated in NHIE.

## 5 CONCLUSIONS

Overall, the expression of SAMSN1 was upregulated in neonatal rats following HI‐induced neurological injury and in cultured cells after OGD. Moreover, inhibition of SAMSN1 could significantly promote cell viability and cell survival with OGD. Our data revealed in NHIE, that suppression of SAMSN1 contributes to neuroprotection, which provides a potential theoretical basis for further research and treatment on NHIE.

## AUTHOR CONTRIBUTIONS

Yi‐Bo Wang contributed to the conceptualization, methodology, investigation, validation, writing original draft, review, and editing. Zong‐Jin Gan contributed to the statistical formal analysis and investigation, visualization. Jun‐Yan Zhang contributed to the statistical formal analysis and investigation, visualization. Somjit Wanchana contributed to software, supervision, and resources. Xi‐Liang Guo contributed to conceptualization, funding acquisition, methodology, project administration, and data curation.

## CONFLICT OF INTEREST

The authors declare no conflict of interest.

## ETHICS STATEMENT

The cortical brain tissues were harvested from a naturally aborted fetus provided by the First Affiliated Hospital of Kunming Medical University, which was approved by the Ethics Committee of Kunming Medical University on September 30, 2015 (approval number: 2015‐9). The rats were fed following the guideline of the China Laboratory Animal Protection and Ethics Committee. The study was approved by the Ethics Committee of Kunming Medical University and the Animal Care & Welfare Committee of Kunming Medical University (no. KMMU2020001) and followed the Guide for the Care and Use of Laboratory Animals published by the US National Institutes of Health.

## Data Availability

The data that support the findings of this study are available on request from the corresponding author.
